# IND-2, a Quinoline Derivative, Inhibits the Proliferation of Prostate Cancer Cells by Inducing Oxidative Stress, Apoptosis and Inhibiting Topoisomerase II

**DOI:** 10.3390/life12111879

**Published:** 2022-11-14

**Authors:** Swapnaa Balaji, Rabin Neupane, Saloni Malla, Rahul Khupse, Haneen Amawi, Shikha Kumari, Diwakar Bastihalli Tukaramrao, Srestha Chattopadhyay, Charles R. Ashby, Sai H. S. Boddu, Chandrabose Karthikeyan, Piyush Trivedi, Dayanidhi Raman, Amit K. Tiwari

**Affiliations:** 1 Department of Pharmacology and Experimental Therapeutics, College of Pharmacy & Pharmaceutical Sciences, University of Toledo, Toledo, OH 43614, USA; 2 Department of Pharmaceutical Sciences, College of Pharmacy, University of Findlay, Findlay, OH 43551, USA; 3 Department of Pharmacy Practice, Faculty of Pharmacy, Yarmouk University, P.O. Box 566, Irbid 21163, Jordan; 4 Department of Pharmaceutical Sciences, College of Pharmacy & Pharmaceutical Sciences, St. John’s University, New York, NY 11432, USA; 5 College of Pharmacy and Health Sciences, Ajman University, Ajman P.O. Box 346, United Arab Emirates; 6 Centre of Medical and Bio-allied Health Sciences Research, Ajman University, Ajman P.O. Box 346, United Arab Emirates; 7 Department of Pharmacy, Indira Gandhi National Tribal University, Lalpur, Amarkantak 484887, Madhya Pradesh, India; 8 Center for Innovation and Translational Research, Poona College of Pharmacy, Bharati Vidyapeeth, Pune 411038, Maharashtra, India; 9 Department of Cancer Biology, College of Medicine and Life Sciences, University of Toledo, Toledo, OH 43614, USA

**Keywords:** prostate cancer, apoptosis, quinoline derivative, IND-2, oxidative stress, mitotic catastrophe, natural drug discovery

## Abstract

In men, prostate cancer (PC) is the most frequently diagnosed cancer, causing an estimated 375,000 deaths globally. Currently, existing therapies for the treatment of PC, notably metastatic cases, have limited efficacy due to drug resistance and problematic adverse effects. Therefore, it is imperative to discover and develop novel drugs for treating PC that are efficacious and do not produce intolerable adverse or toxic effects. Condensed quinolines are naturally occurring anticancer compounds. In this study, we determined the in vitro efficacy of IND-2 (4-chloro-2-methylpyrimido[1″,2″:1,5]pyrazolo[3,4-b]quinolone) in the PC lines, PC-3 and DU-145. IND-2 significantly inhibited the proliferation of PC-3 and DU-145, with IC_50_ values of 3 µM and 3.5 µM, respectively. The incubation of PC-3 cells with 5 and 10 µM of IND-2 caused the loss of the mitochondrial membrane potential in PC-3 cells. Furthermore, IND-2, at 5 µM, increased the expression of cleaved caspase-3, cleaved caspase-7 and cleaved poly (ADP-ribose) polymerase (PARP). The incubation of PC-3 cells with 5 µM of IND-2 significantly decreased the expression of the apoptotic protein, B-cell lymphoma 2 (Bcl-2). Furthermore, 5 and 10 µM of IND-2 produced morphological changes in PC-3 cells characteristic of apoptosis. Interestingly, IND-2 (2.5, 5 and 10 µM) also induced mitotic catastrophe in PC-3 cells, characterized by the accumulation of multinuclei. The incubation of DU-145 cells with 1.25 and 5 μM of IND-2 significantly increased the levels of reactive oxygen species (ROS). Finally, IND-2, at 10 μM, inhibited the catalytic activity of topoisomerase IIα. Overall, our findings suggest that IND-2 could be a potential lead compound for the development of more efficacious compounds for the treatment of PC.

## 1. Introduction

Globally, prostate cancer (PC) ranks among the top five causes of mortality and each year, 1.6 million men are diagnosed with PC and 375,000 will die of PC [1] Primary PC can metastasize to the bones, producing osteolytic and/or osteoblastic lesions and develop into an aggressive form of PC, known as metastatic castration-resistant prostate cancer (mCRPC) [2,3,4]. Furthermore, epithelial to mesenchymal transition (EMT) signaling has been shown to play a critical role in the development of mCRPC [5,6].

Currently, standard androgen deprivation therapy (ADT) and docetaxel, approved by Food and Drug Administration (FDA) in 2004, are the most frequently prescribed first-line treatments for PC [7,8]. However, clinical studies indicate that 20% of patients diagnosed with PC develop resistance to androgen-deprivation therapy and standard chemotherapy [9]. Since 2010, the FDA has approved drugs for the treatment of mCRPC, including cabazitaxel, abiraterone acetate, enzalutamide, apalutamide and darolutamide [8]. These drugs have been shown to be efficacious in treating primary PC; however, the adverse/toxic effects produced by these drugs decrease patient survival and quality of life [8]. The complex biology and heterogenicity of PC tumors suggest that a therapy focused or targeted to a single molecular pathway would be predicted to be less efficacious and consequently, novel compounds that affect multiple oncogenic molecular signals could be developed [10,11]. 

Nature-derived heterocyclic scaffolds, including quinoline scaffolds, have been used to synthesize compounds that have efficacy in treating pain, inflammation, cancer and bacterial infections, among others [12]. Mechanistically, the quinoline compounds have been shown to target topoisomerases, epigenetic enzymes, transcription factors, kinases and microtubules in cancer cells [13]. In order to obtain potent anticancer efficacy, condensed quinoline systems are preferred [14]. For example, a naturally occurring quinoline derivative, camptothecin, which has potent anti-tumor efficacy, is used clinically to treat certain types of cancer [14,15]. Other naturally occurring condensed quinoline systems that have anti-tumor efficacy include cryptoleptine, neocryptoleptine and luotonin A [14]. In 2012, cabozantinib, a quinoline-based, small molecule multi-tyrosine-kinase inhibitor, was approved by the United States Food and Drug Administration (U.S. FDA) to treat several cancer types, including metastatic medullary thyroid cancer, advanced renal cell carcinoma, and hepatocellular carcinoma [16].

Previously, our research group reported the design, synthesis and discovery of IND-2, which has a structural scaffold similar to that of the aforementioned condensed quinoline systems. Our initial findings suggested that IND-2 was efficacious in colon and PC cells. Previously, we described how IND-2 inhibited the proliferation of colon cancer cells, with minimal toxicity in normal cells, and that it did not interact with cellular multidrug efflux transporters [14]. In this study, we conducted extensive experiments to determine the efficacy and mechanism of action of IND-2 in PC cells. 

## 2. Results

### 2.1. The Effect of IND-2 on the Proliferation and Colony Formation of Prostate Cancer Cells 

**Figure 1:**
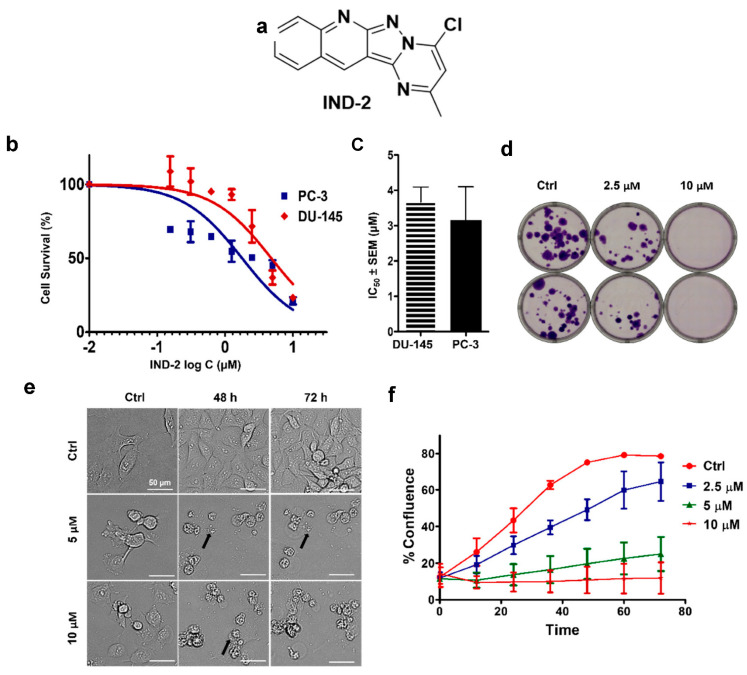
The effect of (**a**) IND2 on the proliferation of (**b**) DU145 and PC3. (**c**) IC_50_ values for PC3 and DU145 cells. The results are represented as the means ± SEM of three independent experiments performed in triplicate. (**d**) The effect of IND-2 (2.5 and 10 µM) on the colony formation rate in PC3 cells. (**e**) Morphological changes induced by IND-2 on PC3 cells after incubation with vehicle and IND-2 (5 and 10 µM) for 0, 48 and 72 h. Biotek Cytation 7 was used to capture the images. (**f**) A representative confluence curve for PC-3 cells incubated with vehicle and IND-2 (0, 1.25, 2.5, 5 or 10 µM).

In vitro, IND-2 (Figure 1a) significantly inhibited the proliferation of PC-3 and DU-145 PC cells, with IC_50_ values of 3 and 3.5 µM, respectively (Figure 1b,c). Furthermore, the colony formation assay was used to determine the inhibitory effect of IND-2 on the proliferation rate of PC-3 cells. The results indicated that at 10 µM, IND-2 significantly decreased colony formation compared to vehicle and 2.5 µM of IND-2 (Figure 1d). Incubation with IND-2, at 5 or 10 µM, significantly decreased the number of viable and adherent PC-3 cells, compared to cells incubated with vehicle (Figure 1e). IND-2 induced a time dependent decrease in the number of viable PC-3 cells, compared to vehicle (Figure 1f).

#### IND-2 Induces Apoptosis in PC-3 Prostate Cancer Cells

Apoptosis is a physiological process that is crucial for maintaining homeostasis in multicellular organisms [17,18]. However, numerous studies indicate that cancer cells can become resistant to certain anti-cancer drugs by evading apoptosis [19,20]. The induction of cellular apoptosis produces chromatin condensation, nuclear fragmentation, nuclear and cytoplasmic condensation, cellular rounding, membrane blebbing, ultrastructural modification of cytoplasmic organelles, loss of membrane integrity and finally, the formation of apoptotic bodies [17,21].

The incubation of PC-3 cells with 5 or 10 µM of IND-2 for 72 h induced shrinkage, rounding and the rupture of PC-3 cells (indicated by black arrows), as shown in Figure 1e. Live cell imaging indicated that 5 or 10 µM of IND-2 increased the number of non-viable or dead PC-3 cells, compared to PC cells incubated with vehicle control (Figure 1f).

### 2.2. IND-2 Significantly Alters the Mitochondrial Membrane Potential of PC-3 Cells

**Figure 2:**
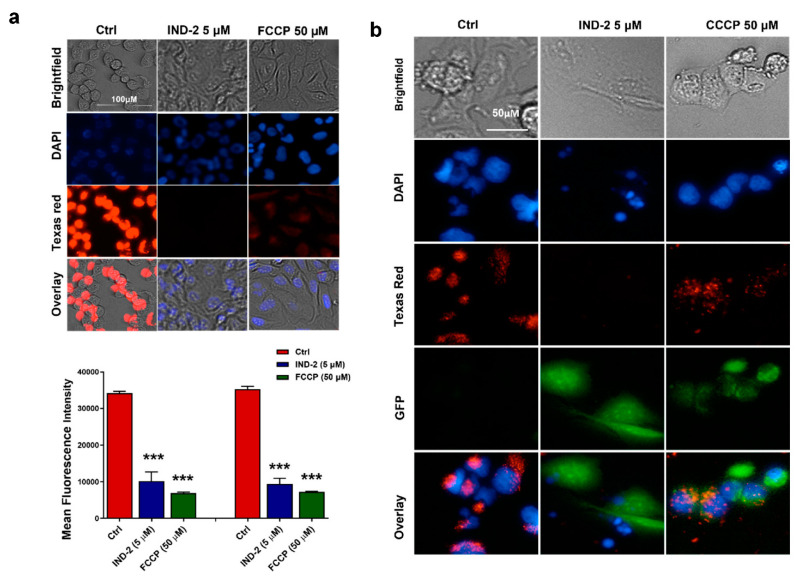
(**a**) IND-2 alters the mitochondrial membrane potential (MOMP) of prostate cancer PC-3 cells. Tetramethyl rhodamine ester (TMRE), is a red dye that is used to stain active mitochondria. PC-3 cells were incubated with vehicle (negative control), IND-2 (5 μM) and the positive control, FCCP (50 μM). IND-2 (5 μM) significantly decreased the MOMP (ΔΨm), compared to the negative control (*** *p* < 0.01). Values represent the mean fluorescence intensities recorded for two independent experiments. Statistical analysis was performed using a two-way ANOVA, with Bonferroni’s multiple comparison post-test. (**b**) The effect of IND-2 on MOMP (ΔΨm) was further assessed using the dye, JC-1. Carbonyl cyanide m-chlorophenyl hydrazine(CCCP) (50 μM) was used as the positive control. Cells were incubated with TMRE dye as shown to validate the results obtained with JC-1. IND-2 (5 μM) decreased the MOMP (ΔΨm), as indicated by a loss of the fluorescence signal from TMRE and a loss of red fluorescence from JC-1.

Mitochondria play a significant role in the regulation of apoptosis [22]. A loss of mitochondrial membrane potential, following the activation of executioner caspases, is one of the earliest steps in apoptosis [23]. Therefore, we determined the effect of IND-2 on the mitochondrial membrane potential. This was done using the tetramethyl rhodamine ester (TMRE) assay. TMRE is a red fluorescent dye that readily accumulates in viable mitochondria due to their negative charge [24]. IND-2, at 5 µM, significantly decreased (*p* < 0.001) fluorescent intensity in PC-3 cells, compared to cells incubated with vehicle, indicating the loss of viable mitochondria due to the loss of mitochondrial membrane potential (ΔΨm) (Figure 2a,b) Carbonyl cyanide p-trifluoro-methoxyphenyl hydrazone (FCCP) is an inducer of plasma membrane depolarization in many types of cells from different species, which disrupts the mitochondrial membrane potential [25]. Consequently, we used this compound as a positive control (Figure 2a,b). Our findings suggest that IND-2 produces, in part, its anti-cancer efficacy by decreasing the number of viable mitochondria and the loss of the mitochondria membrane potential, thus increasing the likelihood of apoptosis in PC-3 cells. These data were further validated using another dye, JC-1. The decrease in the ratio of red to green fluorescence for JC-1 indicates a loss of mitochondrial membrane potential. In PC-3 cells incubated with vehicle the mitochondria appear red with minimal green fluorescence, whereas in cells incubated with IND-2 and the positive control compound, FCCP, the level of green fluorescence increased and the level of red fluorescence decreased, indicating that IND-2 causes the loss of the mitochondrial membrane potential. 

### 2.3. Effect of IND-2 on the Cell Cycle of PC-3 Cells

**Figure 3:**
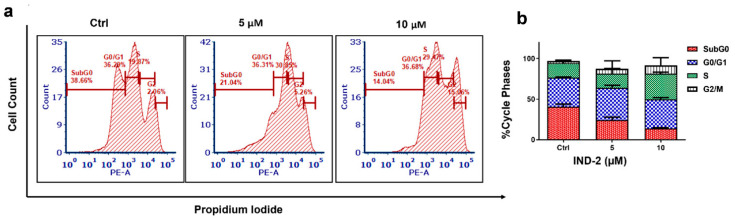
(**a**) In PC-3 cells, the effect of IND-2 (0, 5 and 10 µM) on cell cycle was assessed by flow cytometry (PI, propidium iodide, on the ordinate, and cell count on the abcissa). (**b**) A graph showing the percent change in PC-3 cell cycle for each phase, following incubation with vehicle or 5 and 10 µM of IND-2.

The majority of clinically approved chemotherapeutic drugs induce cancer cell death by apoptosis [26,27,28]. Furthermore, anti-cancer drugs produce cell cycle arrest and inhibit the checkpoint signaling pathways in cancer cells [29]. Thus, we determined the effect of IND-2 on the cell cycle in PC-3 cells. The incubation of PC-3 cells with vehicle (negative control) produced the following distribution in the different phases of the cell cycle: 38.66% in the sub-G0 phase, 36.29% in the G0/G1 phase, 19.87% in the S phase and 2.06% in the G2 phase. (Figure 3a). After the incubation of PC-3 cells with 5 and 10 µM of IND-2, the population of cells in the G2 phase increased to 5.26% and 15.06%, respectively, compared to cells incubated with vehicle. Overall, our results indicated that IND-2 produced significant cell cycle arrest in the G2 phase (Figure 3b). 

### 2.4. IND-2 Induces Nuclear Condensation and Mitotic Catastrophe in PC-3 Cells

**Figure 4:**
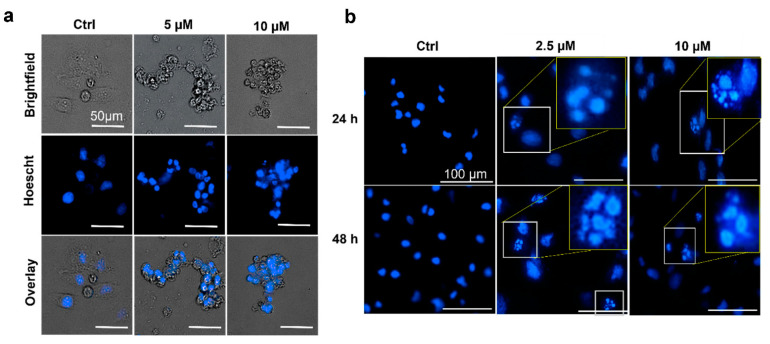
IND-2 induces nuclear morphological alterations in PC-3 cells. Representative images of (**a**) nuclear condensation in PC-3 cells after incubation with 5 and 10 μM IND-2 for 24 h. Live cell imaging was performed using Biotek Cytation 7 and nuclei were stained using Hoescht dye (**b**) IND-2 induces mitotic catastrophe in PC-3 cells 24 and 48 h after the cells were incubated with IND-2 (2.5 and 10 μM). The images were captured using Biotek Cytation 7 and nuclei were stained using DAPI.

Morphologically, apoptosis induces cellular alterations, characterized by condensation and fragmentation of nuclei and cells and the fragmentation of chromosomal DNA into nucleosomal units [20,30]. Cell shrinkage occurs at the onset of apoptosis, followed by the condensation of the nucleus and nuclear chromatin that become highly delineated masses marginated against the nuclear membranes [17]. We determined the effects of IND-2 on the nuclear morphology of PC-3 cells. The incubation of PC-3 cells with Hoescht dye, followed by incubation with 5 and 10 µM of IND-2, indicated the presence of nuclear condensation and fragmentation (Figure 4a). In contrast, incubating PC-3 cells with vehicle did not produce nuclear condensation and fragmentation. (Figure 4a). 

Mitotic catastrophe (MC) occurs when cells fail to undergo mitosis, causing the cells to undergo apoptosis, non-apoptotic death or senescence [31]. The 2012 Nomenclature Committee on Cell Death (NCCD) stated that MC is activated during the M phase by damaging or disrupting the mitotic apparatus, producing mitotic arrest that causes cell death or senescence [31]. MC has been postulated to occur before the onset of apoptosis [31].

Morphologically, MC is characterized by the formation of micronuclei that arise from the nuclear envelope around mis-segregated chromosomal clusters that are one-third of the size of a normal nucleus [31,32,33]. A number of anti-cancer drugs, including cisplatin, taxanes, doxorubicin and etoposide, have been reported to induce cancer cell death by producing MC [31]. Furthermore, MC produces the formation of large cells with multiple micronuclei which induces apoptotic morphology [34]. The incubation of PC-3 cells with 2.5 µM of IND-2 produced large cells with multiple micronuclei, which did not occur in cells incubated with vehicle (Figure 4b). The incubation of PC-3 cells with 5 and 10 µM of IND-2 produces several multinucleated cells similar to that of 2.5 µM of IND-2. (Figure 4b). The induction of MC has been reported to overcome resistance of cancer cells to drugs that induce apoptosis [35]. Therefore, the induction of MC by IND-2 could overcome resistance to apoptosis in cancer cells [35].

### 2.5. IND-2 Activates the Apoptotic Pathway and Induces DNA Fragmentation

**Figure 5:**
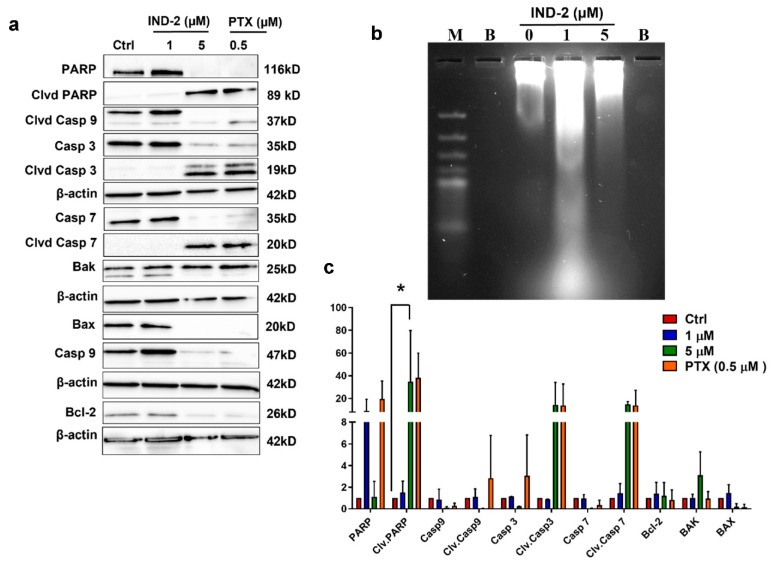
IND-2 induces apoptosis in PC-3 prostate cancer cells. (**a**) Western blots for the proteins involved in the induction of apoptosis, cleaved caspases 3/7 and cleaved PARP, from total cell lysates incubated with vehicle (negative control), IND-2 (1 and 5 µM) and paclitaxel (0.5 µM), for 24 h. (**b**) DNA ladder assay was performed using agarose gel electrophoresis to visualize DNA fragmentation which preceded apoptotic morphological changes. Cells incubated with IND-2 (1 and 5 µM) were harvested and subjected to DMSO DNA extraction. Two thousand ng DNA was run on a 1.5% agarose gel and visualized for fragmentation. IND-2 induced DNA fragmentation as early as 8 h post-incubation ‘B’ indicates blank wells. (**c**) A histogram summarizing the proteins quantified from the western blots using Image J. The data represent means ± SD of two independent experiments (* *p* < 0.05).

Caspases are an evolutionarily conserved family of cysteine—dependent endoproteases that play an essential role in the induction of apoptosis [36,37,38]. For example, caspase-3 and caspase-7, known as the executioner caspases, produce the morphological changes associated with the induction of apoptosis [36]. The activation of caspase-3 and caspase-7 increases the expression of the cleaved forms of caspase-3 and caspase-7, resulting in induction of apoptosis [39]. We determined the expression levels of apoptotic proteins in PC-3 cells after incubation with 1 and 5 µM of IND-2. IND-2 produced a concentration-dependent increase in the expression of cleaved caspase-3 and caspase-7, compared to cells incubated with vehicle (Figure 5a,c). The activation of caspase-3 and caspase-7 by 1 and 5 µM of IND-2 induced the cleavage of the enzyme, Poly(ADP-ribose) polymerase (PARP-1), in a concentration-dependent manner and the cleavage of PARP has been shown to be hallmark characteristic of apoptotic cell death [40,41]. (Figure 5a,c). The Bcl-2 protein family can produce apoptosis by altering MOMP and the subsequent activation of downstream caspase-3 and caspase-7 [42,43]. The Bcl-2 gene is overexpressed in a wide variety of human cancers [44]. Furthermore, the overexpression of the Bcl-2 gene is positively correlated with anticancer drug resistance and efficacy of many clinically used anti-cancer drugs is decreased [44]. Bcl-2 produces anticancer drug resistance by decreasing the probability of apoptosis [44]. Thus, the expression of the Bcl-2 gene and protein represent a major therapeutic target for the design of anti-cancer drugs. The incubation of PC-3 cells with 5 µM of IND-2 decreased the levels of Bcl-2, compared to cells incubated with vehicle (Figure 5a,c). The proteins, Bcl-2 Antagonist/Killer 1 (BAK) and Bcl-Associated X protein, (BAX), are pro-apoptotic proteins that increase the probability of apoptosis [45]. The expression of the BAK gene in PC-3 cells was not significantly altered by 1 or 5 µM of IND-2, compared to cells incubated with vehicle. Interestingly, we saw BAX downregulation after incubation with IND-2 that was comparable to that of the positive control. The loss of BAX and Bcl-2 expression has not been reported before and it remains to be determined what mechanism produces this dual loss. It appears, however, that BAX forms heterodimers with the protein, Myeloid Cell Leukemia 1 (MCL-1), which prevents apoptosis from occurring [46,47]. It remains to be elucidated how IND-2 modulates MCL-1 and regulates Bcl-2 and BAX. Based on our in vitro results, IND-2 increases the probability of cellular apoptosis by altering the expression of several key apoptotic proteins (Figure 5a,c).

DNA fragmentation is one of the hallmarks of apoptotic cell death [48,49]. Therefore, we conducted experiments to determine if IND-2 produces DNA fragmentation. The incubation of PC-3 cells for 8 or 12 h with vehicle (negative control) did not produce DNA fragmentation (Figure 5b). However, the incubation of cells with 1 or 5 µM of IND-2 for 8 or 12 h significantly increased DNA fragmentation compared to cells incubated with vehicle (Figure 5b). 

### 2.6. IND-2 Induces Oxidative Stress in DU-145 Cells

**Figure 6:**
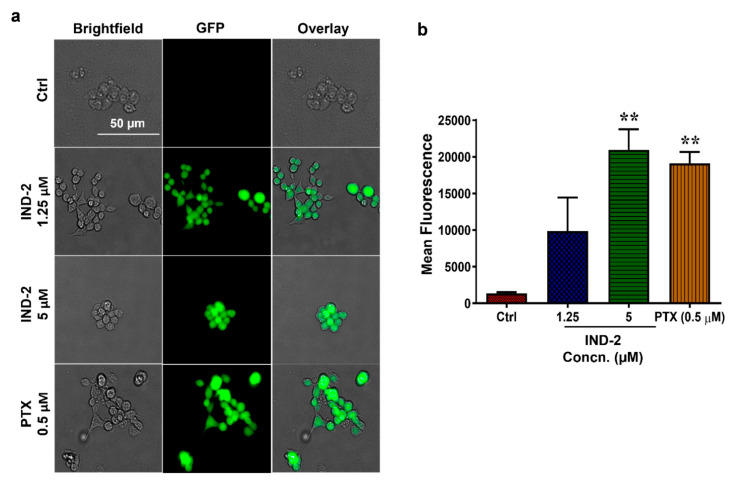
IND-2 induces oxidative stress and the release of ROS in DU-145 cells. (**a**) Representative images of DCF fluorescence levels after incubation with 1.25 and 5 μM IND-2, respectively for 24 h. IND-2 (5 μM) significantly increased the levels of ROS in DU-145 (** *p* < 0.01). Images were obtained using Biotek Cytation 7 Gen 5. Experiments were repeated in triplicates. (**b**) Statistical analysis was performed using One-way ANOVA with Dunnett’s multiple comparison test.

Cancer cells have an antioxidant system that protects them from damage due to the presence of low-moderate levels of intracellular reactive oxygen species (ROS) [50,51]. However, high levels of ROS can surmount the antioxidant system, causing cancer cell death [50]. It has been hypothesized that chemotherapeutic amplification of ROS levels in cancer cells should increase the probability of cell death [52]. Several clinically approved chemotherapeutic drugs can induce oxidative stress in cancer cells [51]. Specifically, anthracyclines, such as daunorubicin, doxorubicin, and epirubicin, increase ROS production in cancer cells [52,53]. Other drugs, such as alkylating agents, platinum coordination complexes, camptothecins, arsenic-based drugs and topoisomerase inhibitors, also induce ROS [52]. As ROS levels increase, the p53 enzyme system becomes activated, and apoptosis is upregulated by increasing transcription of pro-apoptotic proteins, such as BAX and BAK, and by decreasing the transcription of the pro-survival proteins, such as Bcl-2, B-cell lymphoma extra-large (BCL-XL) and MCL-1 [54]. Furthermore, p53, a tumor suppressing protein, increases mitochondrial membrane permeabilization, allowing cytochrome c to be released, an apoptotic molecule, thus increasing the probability of cell death [55]. As a result, we determined whether IND-2 affected ROS levels in DU-145 cells. The ROS levels were determined using 2, 7-dichlorodihydrofluorescein diacetate (H2DCFDA), which is hydrolyzed by esterase enzymes when entering cells, resulting in the formation of H2DCF, which is then oxidized by ROS within the cells into dichlorofluorescein (DCF) [56]. DCF is a highly fluorescent compound and its fluorescence intensity is positively correlated with the intracellular levels of ROS levels [56]. The incubation of DU-145 with 5 µM of IND-2 and 1 µM of paclitaxel produced a significantly greater level of fluorescence, compared to the vehicle control; *p* < 0.001 (Figure 6a,b). Our results are consistent with previously reported findings that paclitaxel significantly increased intracellular ROS levels in cancer cells [57,58]. 

### 2.7. IND-2 Significantly Inhibits the In Vitro Invasiveness and the Migration Potential of PC-3 Cells

**Figure 7:**
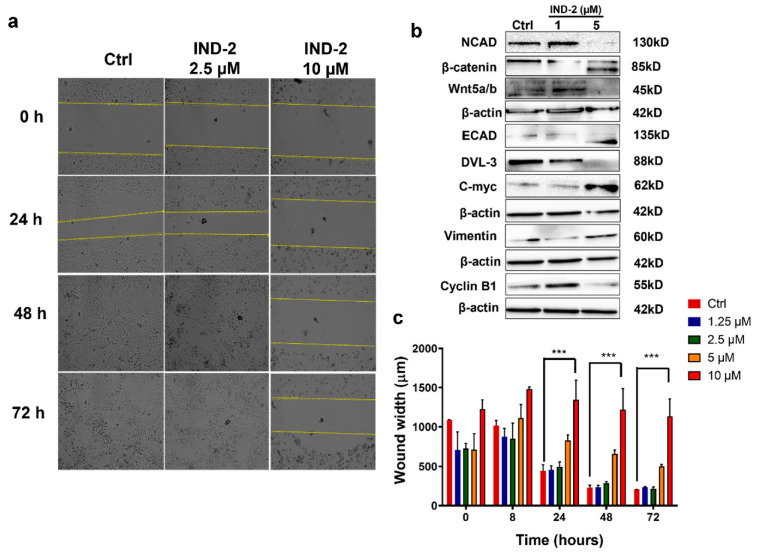
IND-2 has anti-metastatic properties. (**a**,**c**) The in vitro closure of the wound width at various time points (0, 24, 48, 72 h) after incubation with IND-2 (2.5, 5, 10 μM), as observed using Biotek Cytation 7. The wound width was measured using Biotek Cytation 7 Gen 5 and is represented as wound width (μm). The results shown are from two independent experiments (*** *p* < 0.001). (**b**) IND-2 alters the expression of several EMT (Epithelial to mesenchymal transition) markers.

The metastatic progression of cancer is the primary cause of death in patients [59]. Therefore, it is important to develop anti-cancer drugs that prevent cancer cell migration and invasion. To determine whether IND-2 has anti-metastatic efficacy in PC-3 cells, we used an in vitro invasive assay, known as the wound healing assay. IND-2, at 10 μM, significantly inhibited the migration and invasion of PC-3 cells, as early as 24 h, compared to cells incubated with vehicle (Figure 7a,c). The PC-3 cells failed to invade and cover the wound after incubation with 10 μM of IND-2, as compared to cells incubated with vehicle (Figure 7a,c). Furthermore, the wound width remained the same up to 72 h of incubation, starting from 8 h post-incubation (*p* < 0.001) (Figure 7a,c). 

One of the major hallmarks of cancer metastasis is the epithelial to mesenchymal transition (EMT) [60]. The EMT trans-differentiation process confers the transformed epithelial cells with the capacity to invade, resist stress and disseminate [60,61]. In addition, several studies have shown that the Wingless-related integration site (Wnt)/β-catenin signaling pathway may play a key role in EMT [62]. Therefore, we determined the expression of EMT and Wnt/β-catenin markers in PC-3 cells incubated with IND-2. The incubation of PC-3 cells with 5 μM of IND-2 significantly decreased the expression of the mesenchymal marker, N-cadherin and increased the expression of the epithelial marker, E-cadherin, compared to the cells incubated with vehicle (Figure 7b). In addition, IND-2 significantly decreased the expression of Dishevelled Segment Polarity Protein 3 (DVL3), a key regulator of the Wnt/β-catenin pathway (Figure 7b) [63]. β-catenin is a downstream effector of the Wnt/β-catenin pathway [63]. High levels of β-catenin have been reported to increase tumorigenesis [63]. β-catenin induces tumorigenesis by the transactivation of its downstream target genes, cyclin-D1 and C-myc [63]. IND-2, at 5 μM, induced the fragmentation of β-catenin, as indicated by western blotting (Figure 7b), which did not occur in cells incubated with vehicle (Figure 7b). The fragmentation of β-catenin primarily results from activation of apoptosis and plays a role in mediating the anti-proliferative and anti-metastatic efficacy in PC-3 cells [64]. Cyclin B1 is a protein that regulates the initiation of mitosis [65]. The overexpression of cyclin B1 is negatively correlated with a poor prognosis in different types of cancer and is involved in tumorigenesis [65]. Thus, decreasing the levels of cyclin B1 could be a potential approach for treating cancer. The incubation of PC-3 cells with 5 μM of IND-2 significantly decreased the expression of cyclin B1 (Figure 7b). Thus, IND-2 has a significant impact on the Wnt/β-catenin signaling pathway, as well as the expression of several EMT markers. 

### 2.8. IND-2 Inhibits Topoisomerase II Activity

**Figure 8:**
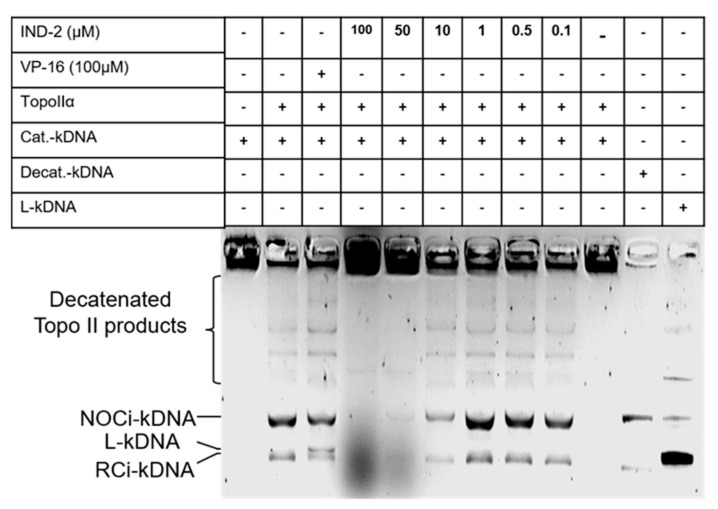
IND-2 inhibits TOPO2 activity and human kinetoplast DNA decatenation. Catenated kinetoplast DNA was incubated with human TOPO2α in presence of the indicated compounds at 37 °C for 30 min. DNA samples were separated by electrophoresis on a 1% agarose gel. The positions of the catenated DNA (F1), decatenated catenated products (FII), and linear DNA (NOCi-kDNA—Non-circular kinetoplast DNA; L-kDNA—Linearized kinetoplast DNA; RCi-kDNA—Relaxed circular kinetoplast DNA.

DNA topoisomerases are enzymes that prevent DNA supercoiling during replication, transcription, recombination and chromatin reorganization [66,67]. Because of the essential roles of these enzymes in the maintenance of cell function, topoisomerases have become targets for the development of anticancer drugs [66]. Since IND-2 shares structural similarity with the quinoline scaffold of many novel topoisomerase II inhibitors [68,69], we conducted a topoisomerase assay to determine if IND-2 inhibits topoisomerase II. IND-2 at 50 and 100 μM, significantly inhibited topoisomerase II activity, compared to cells incubated with vehicle (Figure 8). Decatenation is a critical in vivo reaction of DNA topoisomerases and is an indicator of topoisomerase activity during DNA replication [68] Kinetoplast DNA is frequently used as a substrate in the decatenation assay [70]. In the kinetoplast DNA decatenation assay, IND-2, at 50 and 100 μM, significantly inhibited the activity of Topo IIα. This is in contrast to etoposide, which traps Topo IIα covalently bound to the cleaved DNA strands, inhibiting DNA synthesis [71]. IND-2, at 50 and 100 μM, inhibited topoisomerase IIα from accessing the substrate, kinetoplast DNA (Figure 8). The absence of the decatenated products of topoisomerase IIα, nicked open circular DNA and relaxed circular DNA, after incubation with 50 and 100 µM of IND-2, suggests that IND-2 inhibits the catalytic activity of topoisomerase IIα (Figure 8). Further studies, such as surface plasmon resonance (SPR) and binding studies, will be required to determine if topoisomerase II is a possible target for IND-2.

## 3. Materials and Methods

### 3.1. Chemistry: Synthesis and Characterization of IND-2

The procedure for the synthesis and the analytical data for the compound (IND-2 has been previously reported [14]). 

### 3.2. Biological Studies: Reagents and Dyes

The DMEM (Dulbecco’s modified Eagle’s medium) for cell growth was purchased from GE Healthcare Life Sciences, HyClone Laboratories (Logan, UT, USA). The 2.2 mM EDTA lysis buffer and 0.25% trypsin were obtained from Corning Life Sciences (VWR International, LLC, Radnor, PA, USA). Phosphate buffer saline (PBS) was purchased from Media Tech, Inc. (Manassas, VA, USA). The Fluoroshield mounting medium, together with 6-diamidino-2-phenylindoledihydrochloride (DAPI), was obtained from Abcam (Cambridge, MA, USA). The Alexa Fluor^®^ 488 annexin V was purchased from Thermo Fisher Scientific (Richard St, Wayne, MI, USA). The MTT (dimethylthiazol-2-yl-2, 5-diphenyltetrazolium bromide) reagent was acquired from Calbiochem EMD Millipore (Billerica, MA, USA). We purchased Propidium iodide (PI) from Life Technologies (Eugene, OR, USA). We purchased the 2′,7′-Dichlorofluorescin diacetate powder from Sigma Aldrich (St. Louis, MO, USA 63146).

#### 3.2.1. Cell Line and Culture Conditions

The PC cells, DU-145 and PC-3, were a gift from the late Dr. Gary Kruh, University of Illinois at Chicago. These cells were grown as an adherent monolayer in a culture flask containing DMEM media that was supplemented with 4.5 g of glucose, 10% fetal bovine serum (FBS) and 1% penicillin/streptomycin (in an incubator at 37 °C with 5% CO_2_ and a relative humidity of 95%.

#### 3.2.2. Cytotoxicity Assay

A cell cytotoxicity assay using the MTT assay was performed on PC cells using previously described protocols [26,72]. Briefly, the DU-145 cells were seeded in 96-well plates at a density of 3000 cells per well, and PC-3 cells were seeded in 96-well plates at a density of 2000 cells per well. Immediately following the culturing of the PC cells for 24 h, the cells were incubated for a second 72 h period with IND-2 (at 0.15625, 0.3125, 0.625, 1.25, 2.5, 5, or 10 μM) or vehicle (DMEM media supplemented with 10% FBS and 1% penicillin/streptomycin; control) or the test compound (1–10 μM). Immediately following incubation of the test compound in each well, 20 μL of a solution of MTT reagent at a concentration of 4 mg/mL was added to each well and the cells were incubated for 3 h at 37 degrees Celsius. The media was aspirated, and the formazan crystals that formed were dispersed in 150 μL of DMSO. BioTek™ Synergy™ H1Multi-Mode Reader (Winooski, VT, USA) was used to analyze the 96-well plates at a wavelength of 570 nm.

#### 3.2.3. Colony Formation Assay

The effect of IND-2 on the proliferation of PC cells was determined by measuring the colony formation rate of PC-3 cells after incubation with IND-2. In a 6 well plate, the PC cells were seeded at a density of 250 cells/well and allowed to attach overnight. The following day, cells were incubated with IND-2 (2.5 and 10 μM) and this was done every 3 days for 14 days. On the 14th day, the media was aspirated, and the cells were fixed with methanol. Finally, the cells were incubated with 0.1% crystal violet dye for 20–30 min. The cells were washed vigorously three times with PBS to remove the excess stain and images of the colonies were using the BioTek Citation 7™. 

#### 3.2.4. Live Cell Imaging and Morphological Analysis

The effect of IND-2 on the morphology of PC-3 cells was determined by live cell imaging using BioTek™ Synergy™. Briefly, PC-3 cells were seeded at a density of 3000 cells/well in a 96 well plate. The following day, the cells were incubated with vehicle (negative control) and IND-2 (1.25, 2.5, 5 and 10 μM) and transferred to BioTek Cytation 7™ for live cell imaging. The images were captured at 20-fold magnification for a period of 72 h and the changes in the cell confluence over time was determined based on the images captured at 4-fold magnification. 

#### 3.2.5. Cell Cycle Checkpoint Analysis using Fluorescence-Activated Cell Sorting (FACS)

As described previously [73], a flow cytometric analysis of the cell cycle was performed using propidium iodide (PI) staining to determine the distribution of the cells at each phase of the cell cycle. PC-3 cells were seeded in a 6-well plate at a density of 250,000 cells/well. In a 6-well plate, PC-3 cells were seeded at a density of 250,000 cells per well. Immediately after incubation, the cells were incubated with vehicle or IND-2 at 2.5, and 10 μM for 24 h. Subsequently, the cells were trypsinized with 0.05% trypsin and 2.21 mM EDTA, washed once in phosphate-buffered saline, and resuspended in 1 mL of ice-cold PBS. For staining the cells, 10 µL of the 500 µg/mL PI stock solution was applied. Cells were then incubated on ice for 15 min. The BD FACS Canto™ flow cytometer (BD Biosciences, Becton-Dickinson, San Jose, CA, USA) was used to determine the distribution of the cells in the cell cycle phases (SubG1, G1, S, G2). The data was analyzed using FCS Express 7 cytometry (De Novo software, Pasadena, CA, USA).

#### 3.2.6. Determination of Reactive Oxygen Species (ROS) Levels 

A 96-well plate containing PC-3 cells was seeded at a density of 3000 cells per well and incubated overnight. The adherent cells were treated with either vehicle (DMEM media supplemented with 10% FBS and 1% penicillin/streptomycin; control), IND-2 (1.25 and 5 µM) or the positive control, paclitaxel (0.5 µM) for 24 h. Subsequently, the cells were incubated with 3 µM of DCFDA (2′,7′-dichlorodihydrofluorescein diacetate) for 30 min, followed by gentle washing with ice cold PBS three times. Based on the level of fluorescence generated by the oxidized DCFDA dye, the level of ROS was quantified using BioTek Cytation7™ at a 20-fold magnification. 

#### 3.2.7. Determination of Mitotic Catastrophe

The PC-3 cells were cultured into 6-well plates with microscopic cover slips at a density of 200,000 cells per well and incubated overnight. After adhering for 12 h, the cells were incubated with vehicle (DMEM media supplemented with 10% FBS and 1% penicillin/streptomycin; control) or 2.5 and 10 µM of IND-2. Following incubation for 24 and 48 h, the cells were fixed with 4% paraformaldehyde (1 mL/well) for 15 min. The cells were then gently washed twice with PBS Microscopic coverslips were removed and mounted on glass slides with the cells facing the glass slide in one drop of DAPI (4′,6-diamidino-2-phenylindol) at 1 µM. A fluorescence image of the stained nuclei was obtained using the blue channel in BioTek Cytation 7^TM^ at a 20-fold magnification (DAPI has a maximum absorption wavelength of 358 nm and a maximum emission wavelength of 461 nm). 

#### 3.2.8. Detection of Nuclear Condensation

The nuclear condensation of PC-3 cells following incubation with IND-2 (2.5 and 10 µM) was detected using Hoescht dye, which stains the DNA in the cell nucleus. Briefly, the PC-3 cells were seeded at a density of 3000 cells per well in a 96-well plate. After overnight incubation, the cells were exposed with vehicle (DMEM media supplemented with 10% FBS and 1% penicillin/streptomycin; control) and IND-2 (2.5 and 10 µM). After 24 h of incubation, 1 µM of Hoescht dye was added to the wells and incubated for 20 min. The cells were then imaged on the DAPI channel at 20-fold magnification.

#### 3.2.9. Determination of Mitochondrial Membrane Potential

The induction of apoptosis and changes in mitochondrial membrane potential in PC-3 cells were analyzed using Tetramethylrhodamine, ethyl ester (TMRE) dye. TMRE stains active mitochondria and produces red fluorescence when taken up by active live cells [24]. Briefly, PC-3 cells were seeded at a density of 3000 cells per well in a 96 well plate. After overnight incubation, the cells were exposed with IND-2 at 5 µM and incubated for another 24 h. These cells were then incubated with the positive control, trifluoromethoxy carbonylcyanide phenylhydrazone (FCCP 50 µM) for 1 h. After incubation with IND-2 and FCCP, 1 µL of TMRE dye was added to each well and the cells were imaged on a Texas Red channel using a BioTek Cytation 7™ at 20× magnification. The fluorescence intensity was quantified by measuring the mean fluorescence intensity at excitation/emission wavelength of 549/575 nm. 

The loss of mitochondrial membrane potential was further validated by using the dye, JC-1. Briefly, PC-3 cells were seeded in a 96 well plate at a eedng density of 2000 cells/well. The following day, cells were incubated with IND-2 (5 µM) for 24 h. The following day, cells were incubated with CCCP (50 µM) for 4 h followed by JC-1 (2 µM) and Hoescht (3 µg/mL) and incubated for 30 min. Cells were washed with PBS and imaged using Biotek Cytation 7™ at 20× magnification.

### 3.3. Western Blot Assay for Detecting Apoptotic Proteins and Epithelial to Mesenchymal Transition (EMT) Markers

Western blot assays were used to identify the molecular markers of apoptosis as previously described [74]. Briefly, PC-3 cells were incubated with IND-2 (1 and 5 μM) vehicle (plain media, negative control,) or 0.5 μM of paclitaxel (PTX, positive control) for 24 h. The cells were lysed using the lysis buffer, M-PER™ (ThermoFisher, Rockford, IL, USA) and a protease inhibitor cocktail, containing aprotinin, destatin, E-64, leupeptin, and pepstatin A (Sigma-Aldrich Life Science, St. Louis, MO, USA). The protein concentration of the cell lysates was quantified using the bicinchoninic acid (BCA) assay, as previously described (G-Biosciences, St. Louis, MO, USA) [75]. The lysates were loaded onto a polyacrylamide gel for electrophoretic separation. The proteins were transferred from the gel to a nitrocellulose membrane. The membranes were blocked using 5% milk in Tris-buffer saline Tween 20 (TBST) for 1 h and incubated with primary antibodies (PARP, cleaved PARP, caspase-9, cleaved caspase-9, caspase-3/7, cleaved caspases-3/7, BAK, BAX, BCL-2, β-actin, N-cadherin, E-cadherin, β-catenin, Wnt 5a/b, c-MYC, DVL3, vimentin, and cyclin B1 (1:1000), at 4 °C, with gentle shaking overnight. The membranes were washed with TBST and incubated with horseradish peroxidase labeled (HRP) secondary antibodies (1:3000) for 1 h at room temperature. The membranes were washed and developed using the enhanced chemiluminescent (ECl) substrates, SuperSignal™ Pico/Femto (ThermoFisher, Rockford, IL, USA). ImageJ software was used to quantify the Western blots as previously reported [26] and β-actin was used to normalize the quantified proteins. 

#### 3.3.1. DNA Fragmentation

The DNA extraction and fragmentation assay was used to determine if IND-2 induced apoptosis, as DNA fragmentation precedes the morphological changes produced by apoptosis. Approximately 2 × 106 cells were seeded in 6 mm Petri dishes. The following day, the cells were incubated with vehicle (negative control) and IND-2 (1 and 5 μM). The cells were trypsinzed 8 or 12 h later and washed once with PBS. Additionally, 100 μL of Tris-EDTA (TE) buffer (pH 7.4), with 2% SDS was added to the pellet, followed by mixing and vortexing. Subsequently, the solution was centrifuged at 15,000 rpm for 10 min at 4 °C. DNA was isolated from the supernatant and analyzed using UV (Ultraviolet) spectroscopy by recording the absorbance at 260, 280, and 230 nanometers. Using a ratio of A260/A280, the purity of the isolated DNA was determined. Nucleic acids have an absorbance maximum at 260 nm, and the ratio between this absorbance and the absorbance at 280 nm is used as a measure of purity [76]. After DNA was isolated, it was loaded onto a 1.5% agarose gel and run at 35 V for 6–8 h. An UV light was used to visualize the DNA smear to determine if it was fragmented [77]. 

#### 3.3.2. Wound Healing Assay

The wound healing assay, also known as the scratch assay, was used to evaluate IND-2’s efficacy to prevent invasion and metastasis of the prostate cancer cells. Briefly, PC-3 cells were seeded at a density of 10,000 cells per well in a 96-well plate and allowed to attach overnight. The following day, the cells were observed to determine if they had reached at least 95% confluency and formed a monolayer. If the cells were determined to have formed a monolayer, a scratch was made in the center, using the Incucyte^®^ 96-Well Woundmaker Tool. The cells were immediately incubated with IND-2 (2.5 and 10 μM) and transferred to the BioTek Cytation 7™ to capture live images from (0, 8, 24, 48, 72 h) using the brightfield channel at 4-fold magnification. 

#### 3.3.3. Topoisomerase II Inhibitory Assay 

The assay was done according to the manufacturer’s instructions. Briefly, 100 ng of catenated kinetoplast DNA, 8 units of topoisomerase IIα, together with the test compounds, were incubated for 30 min at 37 °C in 50 mmol/L Tris-HCl (pH 8), 150 mmol/L NaCl, 10 mmol/L MgCl_2_, 0.5 mmol/L Dithiothreitol, 30μg/mL BSA, and 2 mmol/L ATP in final volume of 20 µL of the reaction mixture. The reaction was stopped by the addition of 2 uL of the stop solution (10% SDS) and incubated with 50 µg/mL proteinase K for 15 min at 37 °C. kDNA was electrophoresed on a EB containing 1% agarose gel to separate topoisomers and analyzed using a gel documentation system, G:BOX CHEMI XX9 (Syngene, Frederick, MD, USA).

#### 3.3.4. Statistical Analysis

Data analysis and data representation were performed utilizing the GraphPad Prism 5 software (GraphPad Software, Inc., La Jolla, CA, USA). The IC50 values obtained from the MTT assay were determined using an unpaired t-test with Welch’s correction. Cell cycle distributions and Western blot protein expression quantification were analyzed with two-way ANOVA followed by Bonferroni’s post hoc analysis. In the ROS assay, green fluorescence intensity was analyzed using an unpaired t-test with Welch’s correction.

## 4. Conclusions

Our results indicated that, in vitro, IND-2 significantly inhibited the proliferation of PC cancer cells DU-145 and PC-3 and the formation of PC-3 colonies. Furthermore, IND-2 had anti-metastatic efficacy, based on its decrease in the expression of the EMT proteins, DVL3 and Cyclin B1, and its decrease in the invasiveness and migration of PC-3 cells. Our mechanistic experiments indicated that based on the MTT and colony forming assays and live cell imaging, IND-2 significantly decreased the proliferation of PC-3 and DU-145 cells. Additional experiments indicated that IND-2 could produce its anti-cancer efficacy by inducing oxidative stress, decreasing the mitochondrial membrane potential and inhibiting metastasis by altering the levels of Wnt 5a/b, DVL3, E-cadherin and cyclin B1. Furthermore, SwissADME, an in-silico web tool, has indicated that IND-2 has favorable physicochemical properties, drug-like characteristics and high bioavailability (Supplementary Figure S1) [78]. Overall, our results suggest that IND-2 may represent a promising lead compound for the development of compounds that inhibit the growth, proliferation and metastasis of PC cells.

## Data Availability

Please see attached Supplementary File.

## Supplementary Materials

The following supporting information regarding the physicochemical and pharmacokinetic properties of IND-2 can be downloaded at: https://www.mdpi.com/article/10.3390/life12111879/s1.
