# Supplementary material for: IND-2, a Quinoline Derivative, Inhibits the Proliferation of Prostate Cancer Cells by Inducing Oxidative Stress, Apoptosis and Inhibiting Topoisomerase II
Source: Life (Basel). 2022 Nov 14;12(11):1879. doi: 10.3390/life12111879 (PMC9693996; doi:10.3390/life12111879)

SUPPLEMENTARY INFORMATION

Supplementary Figure S1: The physicochemical properties of IND-2 were evaluated using SwissADME, a free web tool (Daina, et al. 2017) for evaluating pharmacokinetics, drug-likeness, and medicinal chemistry compatibility. The Lipinski rule is not violated by IND-2, which makes it a drug-like molecule. Additionally, it has a favorable bioavailability score of 0.55. This figure was made under the CC-BY 4.0 Creative Commons 4.0 International License.

Figure S1:

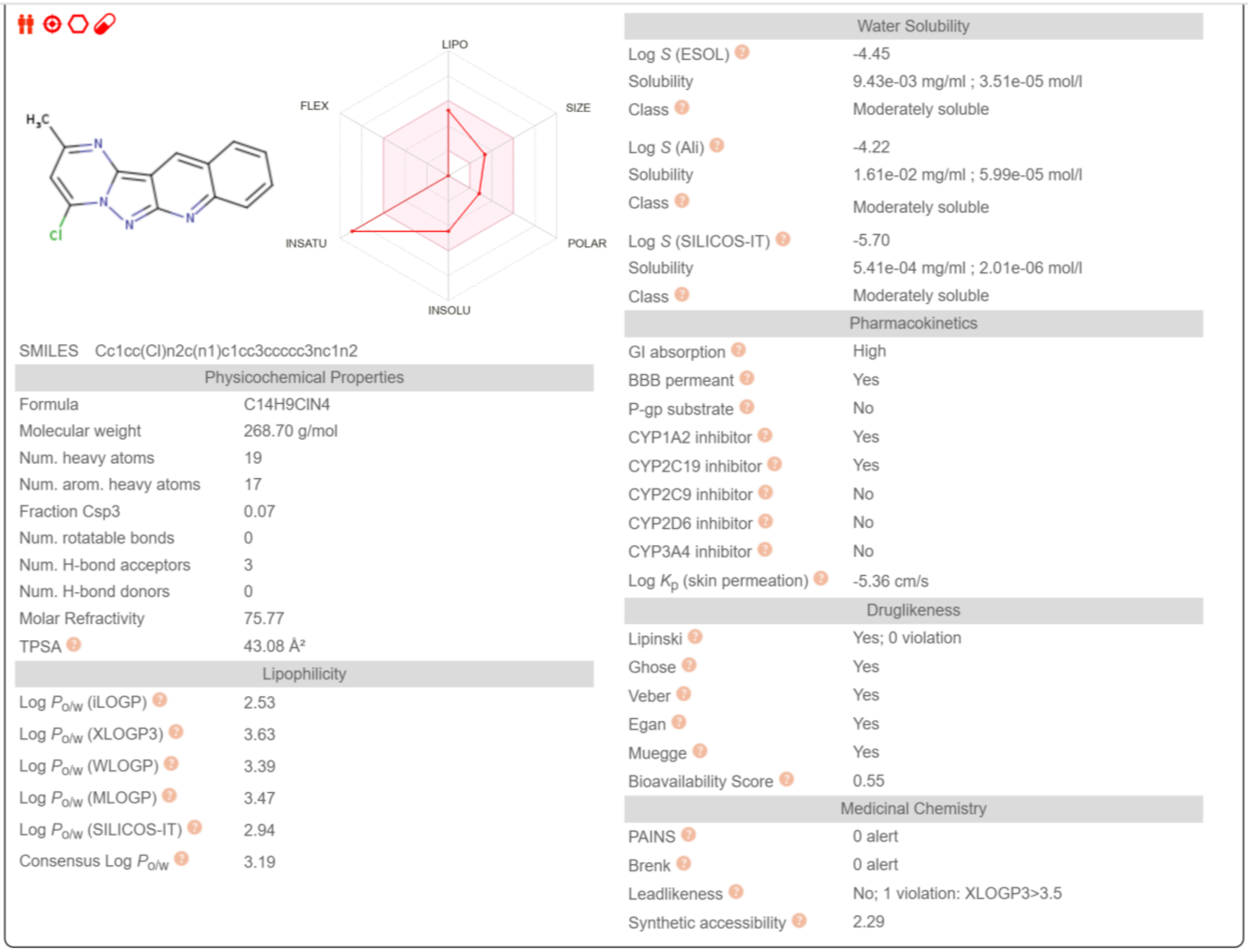

Supplement: Supplementary file 1 [file life-12-01879-s001.zip › life-1961931-supplementary.pdf]
